# Ecological features of trace elements tolerant microbes isolated from sewage sludge of urban wastewater treatment plant

**DOI:** 10.1007/s44154-023-00144-8

**Published:** 2024-01-26

**Authors:** L. Perelomov, V. D. Rajput, M. Gertsen, O. Sizova, I. Perelomova, S. Kozmenko, T. Minkina, Y. Atroshchenko

**Affiliations:** 1 Tula State Lev Tolstoy Pedagogical University (Lev Tolstoy University), Lenin Avenue, 125, Tula, 300026 Russia; 2 Academy of Biology and Biotechnology, Southern Federal University, Rostov-on-Don, 344006 Russia; 3 Federal Research Center “Pushchino Scientific Center for Biological Research of the Russian Academy of Sciences”, G. K. Skryabin Institute of Biochemistry and Physiology of Microorganisms of RAS, Pushchino, 142290 Russia; 4 Tula State University, Lenin Avenue, 92, Tula, 300026 Russia

**Keywords:** Microorganisms, Heavy metals, Metalloids, Wastewater treatment plant, Sewage sludge

## Abstract

Worldwide wastewater treatment plants generate enormous amounts of sewage sludge, and their further disposal depends on the treatment technologies applied and spontaneously occurring microbiological processes. From different ages urban sewage sludge, 12 strains of bacteria with simultaneous tolerance to two or more trace elements: Co, Ni, Cu, Zn, Cd and Pb at concentration of 3-5 mmol were isolated and identified by PCR of target genes and Sanger sequencing methods. The isloated metal(loids) tolerant strains belong to the species, i.e., *Serratia fonticola*, *Rhodococcus qingshengii*, *Pseudomonas fragi*, *Pseudomonas extremaustralis*, *Pseudomonas cedrina*, *Stenotrophomonas maltophilia*, *Serratia liquefaciens* and *Citrobacter freundii*. The ecological features of the isolated strains were studied. The optimal growth temperatures for most strains was 15–30°C at pH range of 5–9, although some strains grew at 7°C (*Pseudomonas fragi* SS0-4, *Serratia fonticola* SS0-9 and *Serratia fonticola* SS12-11). Satisfactory growth of two strains (*Serratia fonticola* SS0-1and *Citrobacter freundii* SS60-12) was noted in an acidic medium at pH 4. Most of the strains grew in the NaCl concentration range of 1–5%. The isolated bacteria resistant to high concentrations of trace elements can be used for the effective mineralization of sewage sludge and for the decontamination of wastewater.

## Introduction

Due to the rapid growth of the world's population and its urbanization, humanity is facing a serious problem of disposal of liquid and solid domestic waste. Over the past decades, the population of the planet has grown faster than ever before. In 1950 there were 2.5·10^9^ people in the world; and in 2022, the planet had 8·10^9^ people. The latest forecasts by the United Nations suggest that population of the Earth could grow to 8.5·10^9^ in 2030, 9.7·10^9^ in 2050 and 10.4·10^9^ in 2100 (United Nations 2022). Most of the population lives in town and cities than in countryside. Approximately 55% of the world’s population residing in urban areas by 2018, and it could increase 68% by 2050 (United Nations 2018).

Sewage sludge (SS) is a byproduct of wastewater treatment and is produced worldwide. In the scientific literature, municipal or urban SS is defined as final solid component from the municipal wastewater treatment process (Grobelak et al. 2019; Nuamah et al. 2012). SS is a complex mixture of organic and inorganic substances of biological and mineral origin. It is a product of physical (after primary treatment), biological (trickling filters, or rotating biological contractors, activated sludge), and physio-chemical (precipitation with lime, alum or ferric chloride) treatment of wastewater (Singh et al. 2022). The main stages of SS processing are thickening, digestion, dewatering and disposal. After wastewater decontamination, the sludge contains large volumes of water. Sludge thickening reduces the volume of water making it suitable for further operations. There are two main strategies for municipal SS management: reuse, including agriculture or landscaping purposes, or final disposal. There are many approaches to reuse SS but also many restrictions on its application (Kacprzak et al. 2017). Anaerobic sludge digestion is a microbiological process where the organic solids in the sludge are transformed to liquids and gases. Final dewatering is done before disposal of sludge that is used as organic fertilizers, sanitary landfill or is incinerated (Roychoudhury et al. 2022).

**Fig 1:**
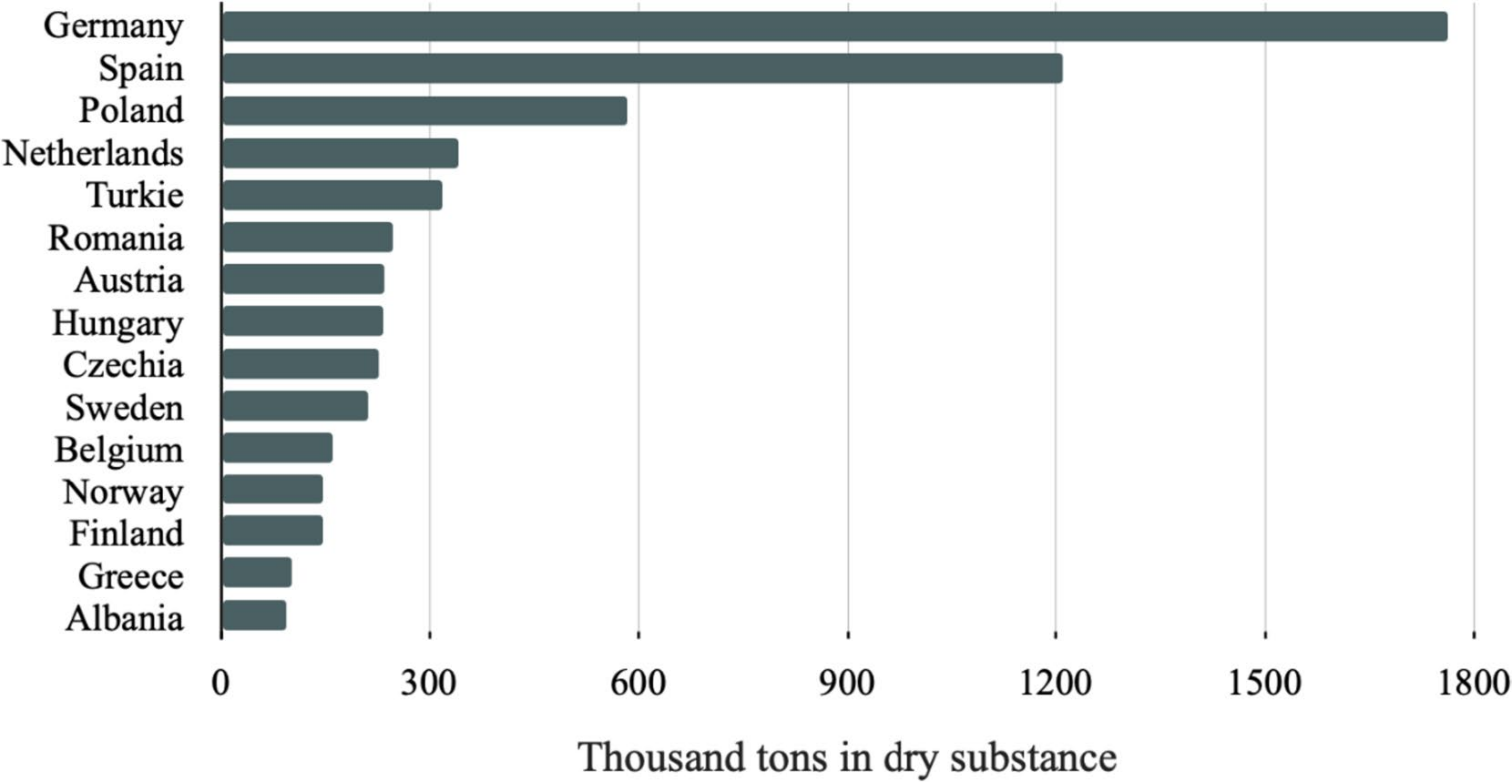
Sewage sludge production and disposal from urban wastewater (thousand tons in dry substance) for some European countries in 2018 (EUROSTAT n.d.)

The sewage treatment plants produce an enormous quantity of SS. Its mass and content are related to the origin of the wastewater and applied technological systems of treatment. The quantity of SS is estimated to be, by mass, on average 3% of the wastewater passing through the treatment plant (Buta et al. 2021). Currently, a production of SS in the world is about 45·10^6^ tons (dry matter) annually (Zhang et al. 2017). North America, Europe and East Asia are main world`s producers of SS (Shaddel et al. 2019). According to the data for 2016, around 30·10^6^ tons of wet sludge with a high moisture content (up to 80%) are produced only in China (Zhang et al. 2017). Urban SS production and disposal for number of European countries in 2018 is shown in Figure 1 (EUROSTAT n.d.) Fig. 1.

SS is characterized by a huge microbial diversity that may affect sludge mineralization, agricultural soil quality at their use as fertilizers and possible contamination of agricultural crops and adjacent environments (Nascimento et al. 2018; Li et al. 2021). Biodiversity of SS may change depending on wastewater origin (industrial or domestic) and conditions of treatment (Cydzik-Kwiatkowska and Zielinska 2016). Chemical features of sludge, such as pH, redox condition, macronutrient content, presence of inorganic and organic pollutants and biological composition of activated sludge can directly impact bacterial communities’ structure (Nascimento et al. 2018). Significant differences in bacterial communities were identified in the samples of inflow and effluent wastewater. Dominant operational taxonomic units varied both spatially and temporally. For example, groups of pathogenic bacteria were sufficiently reduced in effluent water by the treatment process, except for *Leptospira and Legionella* species, which demonstrated a percentage rise from inflow to effluent water (Numberger et al. 2019).

The number of bacterial species in activated sludge at large wastewater treatment plant may varied from 3860 to 4868 (Begmatov et al. 2022; Wu et al. 2019). Communities of wastewater and SS microorganisms include bacteria of different taxonomic (gram-positive and gram-negative), biochemical (nitrification, denitrification, sulfur oxidation, nitrogen fixation, etc.) and physiological (autotrophic, heterotrophic, aerobic, anaerobic) groups, which provide functional benefits for water decontamination, such as removal of different nutrients and pollutants (Ye and Zhang 2013; McLellan et al. 2010). During digestion of sludge by microorganisms (mainly bacteria) organic matter converts into simpler organic substances. Moreover, bacteria are capable of converting inorganic pollutants, such as heavy metals and metalloids, from solution into less mobile organic compounds (own biomass and organic complexes), as well as less soluble inorganic compounds during bioaccumulation, biosorption and biotransformation (Perelomov and Chulin 2014; Priya et al. 2022). So, various microorganisms in sludge significantly reduce the environmental threat from pollutants posed by wastewater (Xie et al. 2021). In addition, microbiological processes largely determine the further use of SSe, which under certain conditions is a valuable organic fertilizer. Sludge microbial communities contain microorganisms of human and animal origin, which also may interact with microorganisms of natural environments including enriching them with genes for resistance to pollutants and antibiotics (Cai et al. 2014). All these microorganisms could deactivate organic and inorganic toxicants only if they are tolerant to high these pollutant concentrations.

**Fig 2:**
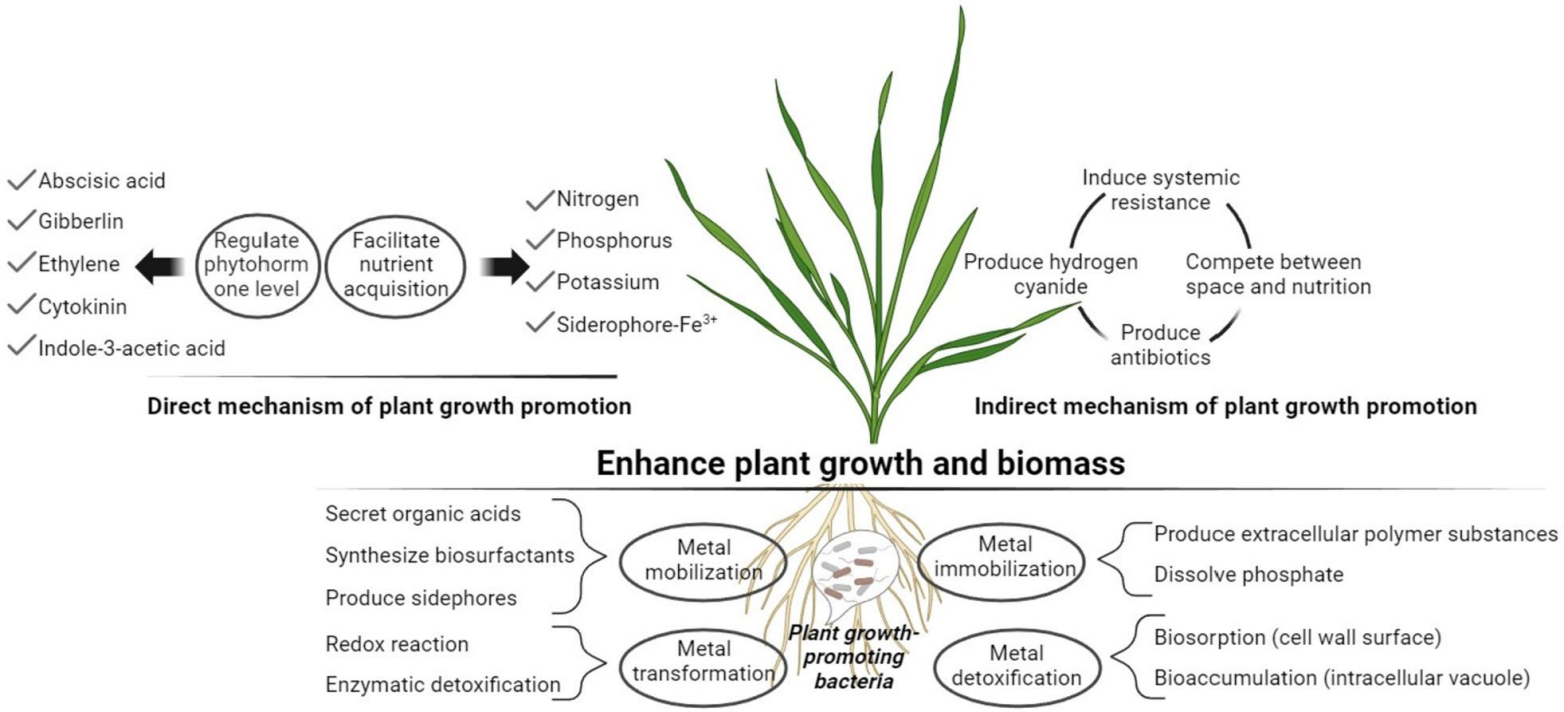
Mchanisms of effects of plant growth-promoting bacteria on the bioremediation of metal-contaminated soils (accoding to Wang et al. 2022)

From an environmental point of view, microorganism strains that are members of activated and SS, tolerant to high concentrations of pollutants, especially heavy metals and metalloids, are of great interest (Perelomov et al. 2022). They are capable of mineralization of SS organic matter and thus reduce their toxicity. Moreover, interaction of metal-tolerant bacteria and plants can play an important role in adaptation of vegetation to soils polluted by metal(loid)s. Different bacteria able to promote plant growth by providing important substances, minimizing the harmful effects of metal(loid)s, as well as boosting tolerance to them (Narayanan and Ma 2023). Various metabolites of plant-associated bacteria (phytohormones such as abscisic acid, cytokinins, jasmonic, ethylene, and gibberellic acids, organic acids, aminoacids, siderophores etc. involve in many processes occurring in the rhizosphere, such as nutrient acquisition, post-embryonic root elongation, metal detoxification and alleviation of biotic/abiotic stress in plants (Rajkumar et al. 2012). Different direct and indirect mechanisms of effects of plant growth-promoting bacteria (PGPB) on the bioremediation of metal-contaminated soils are summarized in Fig. 2 (Wang et al. 2022).

In this investigation, we studied the systematic belonging of some strains of bacteria tolerant to high concentration of trace elements isolated from uneven-aged SS of the urban wastewater treatment plant and ecological conditions (temperature, pH, salinity) for their normal activity.

## Results and discussion

### Isolated and identified bacterial strains in sewage sludge

The humidity of the collected sludge samples was noted between12-18%. The elemental composition (H, C, N, S) of SS of different ages is presented in Table 1.

**Table 1:** Elemental composition and pH of air-dry sewage sludge of different ages (arithmetic mean ± standard deviation)

**Sewage sludge**	**Elements**				**Ratio** **C/N**	**pH**
	**N [%]**	**C [%]**	**H [%]**	**S [%]**		
**Fresh**	2.36±0.098	28.94±0.975	3.68±0.457	1.28±0.092	12.26±0.095	7.94±0.14
**1 month**	2.49±0.031	28.57±0.394	3.40±0.342	1.20±0.065	11.48±0.015	7.73±0.03
**6 months**	2.20±0.154	22.53±1.895	2.71±0.571	1.646±0.026	10.22±0.140	7.71±0.01
**12 months** **(1 year)**	3.26±0.103	33.22±0.541	4.23±0.191	1.45±0.013	10.21±0.160	7.70±0.02
**60 months** **(5 years)**	1.86±0.006	15.91±0.060	2.53±0.156	3.67±0.001	8.58±0.005	7.14±0.01

Undoubtedly, the elemental composition of SS depends on the initial composition of wastewater and the features of the technological process of treatment, which is more reflected in sludge up to 1 year old. However, there is a trend towards a decrease in the content of N, C, H during long-term storage of sediments, and the C/N ratio also decreased (Table 1). The decrease in the content of nitrogen and organic carbon in sludge of five years of age indicates its active mineralization. At the same time, sulfur compounds are relatively stable and accumulate in SS over time.

The content of total heavy metals (Co, Ni, Cu, Zn, Cd, Pb) in SS was given in Table 2.

**Table 2:** Permissible total concentrations of heavy metals and metalloids in sewage sludge (State Standard 2001) and concentrations of heavy metals and metalloids in air dry sewage sludge of different ages (ppm)

**Element**	Concentration, mg/kg of dry matter, not more, for sludge of the group		Concentration of trace elements in sewage sludge of different ages				
	**I**	**II**	**Fresh**	**1 months**	**6 months**	**12 months**	**60 month**
Co	-	-	28.56	28.80	27.94	30.39	309.04
Ni	200	400	66.79	33.30	72.33	50.11	69.00
Cu	750	1500	228.36	171.12	269.24	272.18	102.64
Zn	1750	3500	1119.57	901.36	**1791.15**	**1865.76**	**2360.77**
Cd	15	30	8.66	2.88	10.81	4.04	**32.97**
Pb	250	500	37.99	27.00	37.44	27.62	60.03

The metal concentrations in bold font are those exceeding the maximum permissible concentrations (MPC) for SS of the group I and relating this sludge to the group II. In the Russian Federation, according to State standards, all SS is divided into two groups according to the concentration of trace elements and, accordingly, to the possible nature of use in agriculture. For each group, there are MPC of trace elements (Table 2) (State Standard 2001).

If the content of at least one of the normalized elements exceeds its MPC for group I, then sludge is classified as group II. The SS of group I is used for all types of crops, except for vegetables, mushrooms, greens and strawberries. The sludge of group II is used for cereals, legumes, grain fodder and industrial crops.

The sludge of groups I and II are used in industrial floriculture, green building, forest and decorative nurseries, for biological reclamation of disturbed lands and solid waste landfills. Doses of sludge for agricultural crops in each case are calculated taking into account the actual content of normalized pollutants in sludge and soil. If the soil contains any of the normalized contaminants at a concentration of more than 0.8 MPC, the application of sludge as a fertilizer is prohibited. Thus, the studied SS, starting from 6 months old, can be attributed to the second group due to the excess Zn concentration, and five-year-old sludge - due to the Cd concentration (Table 2).

The number of colony-forming units (CFU) in SS of different ages was: 1.2 × 10^7^ cells/g in fresh SS, 3.1 × 10^7^ cells/g in 6-month-old SS, 3.8 × 10^6^ cells/g in 1-year-old SS, 2.8 × 10^7^ cells/g in 5-year-old SS.

Based on the results of inoculation on the medium with trace elements, bacteria with medium (3 mM) and high (5 mM) resistance to Co, Ni, Cu, Zn, Pb and Cd were isolated (Table 3). Zinc and Pb resistant bacteria were present in all SS samples. As for bacteria resistant to Cd and Co, they were rare (Table 3). Some bacteria were resistant simultaneously to two and more elements, for example, Zn and Cd, Pb and Zn. Such multi-resistant bacteria were of interest to us.

**Table 3:** The presence of resistance to trace elements in bacteria in different samples of sewage sludge

**Sewage sludge of different ages**	**Presence of bacteria resistant to 3 mmol and 5 mmol of the respective trace element**					
	**Co**	**Ni**	**Cu**	**Zn**	**Pb**	**Cd**
**Fresh**	˗˗	+	+	+	+	+
**1 month**	˗˗	˗˗	˗˗	+	+	˗˗
**6 months**	˗˗	+	+	+	+	˗˗
**12 months**	˗˗	+	+	+	+	˗˗
**60 months**	+	+	+	+	+	˗˗

**Fig 3:**
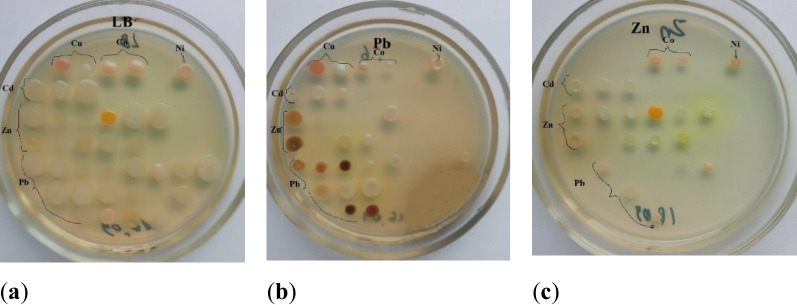
Color change of microorganism colonies under the influence of trace elements: (**a**) - initial colonies of microorganisms; (**b**) - color of colonies grown on medium with Pb; (**c**) - color of colonies grown on medium with Zn

When cultivated on a medium with high trace elements concentrations, a number of bacterial colonies acquired a specific color (Fig. 3).

There are different views as to why microbial pigments form in response to the presence of trace elements. It is possible that the production of a number of pigments can protect microorganisms from photooxidative damage (Armstrong 1994). However, there is often no linear relationship between the color intensity and the metal concentration in the medium, and pigmentation appears only in a certain range of element concentrations (Lima de Silva et al. 2012). Some scientific publications have argued that the ability to produce pigment can be directly related to metal(loids) tolerance. For example, Fujimore et al. (1996) observed that the red pigment-deficient white mutant of the *Pseudomonas* K-62 strain was more sensitive to Hg^2+^ than the original reddish wild-type strain. Melanin acts as a protective agent against oxidizing agents and trace elements (Nosanchuk and Casadevall 2003). A metabolically engineered bacterium was employed to produce blue-purple pigment violacein responsive to toxic Cd^2+^. Visual and quantifiable signals could be captured after a 1.5-h Cd^2+^ exposure (Hui et al. 2022).

The large number of publications were also indicated a great diversity and variation in the structure of bacterial communities in SS. In the conditions of Brazil (Sao Paulo), *Clostridium* was the dominant genera of SS, followed by genera *Treponema, Propionibacterium, Syntrophus,* and *Desulfobulbus* (Nascimento et al. 2018). The SS of Navarra region (Spain) contains in concentrations between 10^5^ and 10^7^ CFU g^−1^ of total coliforms, i.e., *Escherichia coli*, *Staphylococcus aureus*, *Enterococcus* sp., *Pseudomonas* sp. and total mesophilic bacteria and it does not contain *Salmonella* sp. (Miguel et al. 2020). Under the climatic conditions of Croatia, a study of 10 wastewater treatment plants produced results showing that eight groups (*Anaerolineaceae, Methanosaeta, Trichococcus, Acinetobacter, Romboutsia, Clostridium, Turicibacter, Intestinibacter*) were present in all studied urban SS.

### Characteristics of bacterial isolates and their significance

In our research molecular genetic methods have identified 12 metal-tolerant strains of bacteria resistant to two and more trace elements (in concentration 3 mmol, 5 mmol) simultaneously in SS of different ages (Table 4). The isolated metal(loid)-tolerant strains belong to different genera of both gram-negative and gram-positive bacteria.

**Table 4:** Strains of trace elements tolerant bacteria isolated from sewage sludge of different ages

**№**	**Strain**	**Isolated from sewage sludge age**	**Tolerance**
1	*Serratia fonticola* SS0-1	Fresh	5 mmol Cu, 3 mmol Pb
2	*Pseudomonas fragi* SS0-4	Fresh	3 mmol Cd, Zn, Cu, Pb
3	*Stenotrophomonas maltophilia* SS0-5	Fresh	3 mmol Zn, 3 mmol Cu
4	*Pseudomonas extremaustralis* SS0-6	Fresh	5 mmol Zn, 3 mmol Cu, Pb
5	*Serratia fonticola* SS0-9	Fresh	5 mmol Pb, 3 mmol Ni
6	*Stenotrophomonas maltophilia* SS0-10	Fresh	5 mmol Pb, 3 mmol Zn
7	*Rhodococcus qingshengii* SS6-3	6 months	5 mmol Ni, 3 mmol Pb, Cu
8	*Serratia fonticola* SS12-11	12 months	5 mmol Pb, 3 mmol Cu
9	*Rhodococcus qingshengii* SS60-2	60 months	5 mmol Co, 3 mmol Ni, Pb, Cu
10	*Pseudomonas cedrina* SS60-7	60 months	5 mmol Zn, 3 mmol Cu
11	*Serratia liquefaciens* SS60-8	60 months	5 mmol Zn, 3 mmol Cu
12	*Citrobacter freundii* SS60-12	60 months	5 mmol Pb, 3 mmol Zn

The *Rhodococcus qingshengii* SS60-2 strain resistant to Co was isolated from 5-year-old sediments with the maximum concentration of this element (309 mg/kg). *Rhodococcus qingshengii* SS6-3 strain, resistant to Ni, was isolated from SS 6 months old with the maximum concentration of Ni (72.3 mg/kg), although slightly exceeding the concentrations in other SS. Zn-resistant *Pseudomonas cedrina* SS60-7 and *Serratia liquefaciens* SS60-8 strains were isolated from 5-year-old SS with the maximum Zn concentration (2360.8 mg/kg). The *Citrobacter freundii* SS60-12 strain, resistant to Pb, was isolated from 5-year-old SS with the highest metal content (60 mg/kg). Thus, the metal tolerance of isolated strains of bacteria in almost half of the cases correlates with the maximum content of trace elements in their habitat.

The question of the factors that determine the composition and structure of the bacterial community in SS is debatable. Authors, using weighted UniFrac distance-based redundancy analysis, found that a group of 5 trace elements (Cr, Cu, Ni, Pb, Zn) and a pair of organic contaminants (hexabromocyclododecane and tribromodiphenyl ethers) were significantly associated with the bacterial community structure in mesophilic anaerobic stabilized samples. Altogether, 85% of the variance in bacterial community structure could be ascribed to these pollutants (Stiborova et al. 2020). Major et al. (2022) points out that the presence of some trace elements (iron, lead, mercury or zinc) in SS samples seems to be associated with particular microbial community. At the same time, the Nascimento et al. (2018) discovered that inorganic toxicants, such as heavy metals, had little impact on the structure of microbial community in the sludge (Nascimento et al. 2018). The sequencing results of Bhat et al. (2020) also revealed that the bacterial diversity showed evident variations under heavy metal stress. Cluster analysis and Principal component analysis showed that the microbial community in SS with high Cu(II) concentration was different from the sludge samples with high Cd(II) and Pb(II) concentrations. Obviously, the influence of heavy metals and metalloids on the structure of the microbial community depends on various factors, including the concentrations of pollutants and the initial composition of the community. It was also noted that the composition of the microbial community of SS does not correlate with the method of biosolids stabilization (aerobic processing, aerobic plus anaerobic stabilization; and no stabilization and with lime addition) and on the number of residents served by wastewater treatment plants (Major et al. 2022). The high contents of Fe and S were important modulators of microbial community structure for some sludge after anaerobic treatments and with relatively low N and P contents (Nascimento et al. 2018). At the same time high content of these elements was important factor for municipal, aerobically treated sludge with low Fe and Al concentrations. In addition, there can be a substantial seasonal variation in the composition of the microbial community, described in the literature (Numberger et al. 2019).

A detailed examination of the genera and species of strains identified by us shows that all of them were either found earlier in SS or active sludge treatment plants, or are extremophiles.

*Serratia* are facultative anaerobes, catalase-positive, and motile with peritrichous flagella. The bacteria can use many different compounds as carbon sources in medium containing ammonium sulfate as the nitrogen source (Batt and Robinson 2014). *Serratia fonticola* found in a wide array of environments, including drinking water, soil and sewage. For example, *Serratia fonticola* IB4r (NCTC 13193) was isolated from pilot-scale sewage bed (Ashelford et al. 2001).

*Serratia liquefaciens* can be found typically in soil, wastewaters, sewage, but also in food or in the intestinal tract of humans or animals (Liu et al. 2015; Lepesova and Krahulcova 2019). Isolated from contaminated soil bacterium *Serratia liquefaciens* producing lignin peroxidase is able to degrade effluent with substantial reduction of lignin (58%) and phenol (95%) (Haq et al. 2016). The metal tolerance of strains of *Serratia liquefaciens* has been shown in the works of Han et al. (2018), Ahmed et al. (2020) and others.

Fast growth and high adaptability to different ecological conditions (oxidative, nutritional, etc.) allows *Pseudomonas* genus one of the most diverse and ubiquitous group (Peix et al. 2009). Members of *Pseudomonas* are able to mineralization of various organic compounds (aromatic hydrocarbons, chloro- and nitro-organic compounds, pesticides) and play an important role in the bioremediation and detoxification of contaminated soils and grounds. *Pseudomonas spp*. are also key participant of bacterial community active in wastewater treatment processes. In the studied of SS, we found strains that can be attributed to three species: *Pseudomonas fragi, P. cedrina* and *P. extremaustralis*.

The discovery by us of a strain of the *P. extremaustralis* species requires further confirmation by analyzing the full genome of the isolated strain because this species is very rare (López et al. 2017). However, a strain of this species was isolated from wastewater by Spanish researchers (Vargas-Ordóñez et al. 2023). This species isolated from a season pond in Antarctica presents high levels of resistance to oxidative stress and low temperature. It is also resistant to hydrocarbons and able to them degradation (Cai et al. 2014). *P. extremaustralis* resisted to 4 mM Cu^2+^ in a rich microbial medium such as LB (Colonnella et al. 2019). This Cu resistance mechanism includes the presence of the *cus* and *cop* operons together with other efflux systems and porins located in a single region in *P. extremaustralis* genome (López et al. 2017). Cu^2+^ negatively affected on degradation of diesel despite the fact that Cu enhanced bacterial attachment to hydrocarbons. At the same time, when a small amount of glucose (0.05% w/v) was added to the bacteria, the presence of Cu^2+^ intensified degradation of alkanes (Colonnella et al. 2019).

*Pseudomonas fragi*, psychrotrophic bacterium, which has high proteolytic potential (Liu et al. 2015), can produce several types of enzymes, including lipases and proteases. These enzymes are responsible for the spoilage of meat, fish, vegetables, dairy products and other products of animal origin.

*Pseudomonas cedrina* is a rod-shaped bacterium isolated from Lebanese spring waters (Dabboussi et al. 1999). It has been placed in the *P. fluorescens* group, and showed that the species was resistant to high concentrations of NaCl (Tirry et al. 2021).

*Stenotrophomonas maltophilia* is an aerobic, nonfermentative bacillus that is closely related to the *Pseudomonas* species (Calza et al. 2003). Its name means "a unit feeding on few substrates". "Maltophilia" translates from Greek as "affinity, love for malt". It is frequently isolated from soil, water, plant matter, animals, and hospital equipment (Denton and Kerr 1998). This species is found in effluents and rivers that receive sewage water of pig farms (Kim et al. 2018). Strains of *Stenotrophomonas maltophilia* was isolated from anaerobic sludge from sewage treatment plant (Feng et al. 2015). From soil of polluted wasteland novel resistant to Cu bacteria *S. maltophilia* PD2 was isolated (Ghosh and Saha 2013). Pb, Zn and Ni resistant bacterial strains of *S. maltophilia* were isolated from a wastewater treatment plant in Poland (Wierzba 2015).

*Rhodococcus* spp. are isolated from many polluted areas; dominate in hydrocarbon-contaminated ecosystems (van der Geize and Dijkhuizen 2004). *Rhodococci* are capable of utilizing a wide range of organic compounds, including toxic ones (Kawagoe et al. 2019). They are able to metabolize a wide range of pollutants including petroleum hydrocarbons, polychlorinated biphenyls, pharma pollutants, pesticides, explosives, flame retardants, plasticizers, defoliants, dyes, and microplastics. Moreover, the bacteria are resistant to multiple stresses; are able to maintain high metabolic activities under adverse conditions (Krivoruchko et al. 2019; Krivoruchko et al. 2023).

Most strains of *Rhodococcus* have been found to have very high levels of metal resistance. These bacteria are not only capable of metabolizing various organic pollutants in the presence of co-contaminating trace elements, but they able to biosorption and/or bioconversion of various metals and metalloids. Bacterial strains belonging to the *Rhodococcus* genus exploit different mechanisms enabling them to highly tolerate metal(loid) compounds (Pavel et al. 2013). For instance, the reduction of cellular sensitivity, the intracellular sequestration of metal ions and oxyanions, their complexation with siderophores, the alteration of the membrane permeability, mutations, and repairing mechanisms of the DNA responsible for both plasmid and chromosomal DNA stability are some of the mechanisms implemented by bacteria to tolerate and/or resist to metalloids’ toxicity (Stillman and Irwim 1995; Garbisu and Alkorta 2003; Figueira et al. 2006; Presentato et al. 2019).

The species *Rhodococcus qingshengii* was first isolated from the soil contaminated by carbendazim. Although this species is a typical soil inhabitant, it has been found in different habitats such as sludge, sawdust, and seawater (Peng et al. 2023).

*Citrobacter freundii* is a soil microorganism, but can also be found in water, food, the intestinal tracts of animals and humans and sewage (Kus and Burrows 2008). The strain *C. freundii* JPG1, isolated from gold mining tailing in China was cross-tolerate to several trace elements: Ag^+^, Cd^2+^, Co^2+^, Cr^6+^, Cu^2+^ and Ni^2+^ and was able to biosorption and bioaccumulation of Cu under both aerobic and anaerobic conditions (Wang et al. 2018). The strain *C. freundii* SRS1 from Bangladesh could tolerate up to 3 mmol/ L Pb(NO_3_)_2_, 2.5 mmol/ L CoCl_2_, 2.5 mmol/ L Cd(CH_3_COO)_2_, and 2.5 mmol/ L CrCl_3_. The genome of *C. freundii* SRS1 *czcA*, *czcD*, *cbiN*, and *cbiM* genes for Co resistance; *chrA* and *chrB* genes for Cr resistance; and *zntA* gene for Pb and Cd resistance (Uddin et al. 2022).

Thus, numerous literature data confirm the presence of the species identified by us in SS, as well as their metal tolerance.

### Optimal environmental conditions (temperature, pH, salinity) for isolated metal-tolerant strains

An interesting question is whether the isolated trace element tolerant strains are also extremophiles in relation to the most important environmental factors - temperature, pH, salinity, or whether their adaptation to high concentrations of trace elements is due to a specific mechanism. That's why for isolated from SS metal-tolerant species of bacteria, the features of the conditions for their growth were studied – the optimal values of temperature, acidity and salinity of the environment.

The range of optimal growth temperatures for most strains for 48 hours was 15–30°C (Table 5). After 2 days of cultivation, the growth of 3 strains was noted at a temperature of 7°C: *Pseudomonas fragi* SS0-4, *S. fonticola* SS0-9 and *S. fonticola* SS12-11. The same bacterial strains *S. fonticola* SS0-9 and *S. fonticola* SS12-11 as well as strain *Stenotrophomonas maltophilia* SS0-10 showed growth at 37°C. Species of *Serratia* are known to give optimum growth from 20-37°C (Giri et al. 2004). However, some strains of *S. liquefaciens*, *S. odorifera*, *S. plymuthica*, and *S. ficaria* can grow at 4–5°C; other strains of *S. odorifera*, *S. marcescens*, and *S. rubidaea* can grow at 40°C. At the same time, the strain of bacterial species *P. extremaustralis*, which was mostly isolated from places with a cold climate, did not show psychrophilicity in our experiment.

**Table 5:** Growth of isolated strains at different temperatures after 2 days of cultivation

**Strain**	**T of incubation, °C**					
	**7**	**15**	**24**	**30**	**37**	**42**
*Serratia fonticola* SS0-1	-	-	+	+	-	-
*Rhodococcus qingshengii* SS60-2	-	-	+	+	-	-
*Rhodococcus qingshengii* SS6-3	-	+	+	+	-	-
*Pseudomonas fragi* SS0-4	+	+	+	+	-	-
*Stenotrophomonas maltophilia* SS0-5	-	+	+	+	-	-
*Pseudomonas extremaustralis* SS0-6	-	+	+	+	-	-
*Pseudomonas cedrina* SS60-7	-	+	+	+	-	-
*Serratia liquefaciens* SS60-8	-	+	+	+	-	-
*Serratia fonticola* SS0-9	+	+	+	+	+	-
*Stenotrophomonas maltophilia* SS0-10	-	+	+	+	+	-
*Serratia fonticola* SS12-11	+	+	+	+	+	-
*Citrobacter freundii* SS60-12	-	+	+	+	-	-

Cultivation of the studied strains in a liquid LB medium at temperature of 28°C with shaking for 18 h followed by determination of optical density revealed that all strains grew in the pH range of 5–9. Only two strains, *Serratia fonticola* SS0-1and *Citrobacter freundii* SS60-12, grew in an acidic medium at pH 4. After the digesters, wastewater usually has a slightly alkaline reaction. The SS, we have selected was slightly alkaline or close to neutral pH (Table 1). At the same time, with age, the pH of SS is somewhat acidified, changing towards the acidity of zonal soils. It is logical that among the studied bacterial strains there are practically no acidophilic ones.

During cultivation for 2 days at 28°C on LB agar medium with different NaCl salt concentrations in Petri dishes, colony formation was observed for the following strains (Table 6).

**Table 6:** Growth of isolated strains at different NaCl concentrations

**Strain**	**Concentration NaCl, %**								
	**1**	**2**	**3**	**4**	**5**	**6**	**7**	**8**	**10**
*Serratia fonticola* SS0-1	+	+	+	+	+	+	+	-	-
*Rhodococcus qingshengii* SS60-2	+	+	+	+	-	-	-	-	-
*Rhodococcus qingshengii* SS6-3	+	+	+	+	+	-	-	-	-
*Pseudomonas fragi* SS0-4	+	+	+	+	+	-	-	-	-
*Stenotrophomonas maltophilia* SS0-5	+	+	+	+	-	-	-	-	-
*Pseudomonas extremaustralis* SS0-6	+	+	+	+	-	-	-	-	-
*Pseudomonas cedrina* SS60-7	+	+	+	+	+	-	-	-	-
*Serratia liquefaciens* SS60-8	+	+	+	+	+	-	-	-	-
*Serratia fonticola* SS0-9	+	+	+	+	+	-	-	-	-
*Stenotrophomonas maltophilia* SS0-10	+	+	+	+	+	-	-	-	-
*Serratia fonticola* SS12-11	+	+	+	+	+	+	-	-	-
*Citrobacter freundii* SS60-12	+	+	+	+	+	+	+	+	-

Most of the strains grew in the NaCl concentration range of 1–5%, strains (*Serratia fonticola SS0-1*, *Serratia fonticola SS12-11*, *Citrobacter freundii* SS60-12) grew at 6% salt, two strains of them (*Serratia fonticola SS0-1*, *Citrobacter freundii* SS60-12) grew at 7% and one (*Citrobacter freundii* SS60-12) grew at 8% NaCl weakly. It is interesting to note that 7% sodium chloride resistant strains *Serratia fonticola* SS0-1 and *Citrobacter freundii* SS60-12 also grow at low pH (pH 4), and 6% salt resistant strain *Serratia fonticola* SS12-11 also grows successfully at low temperatures (7 ºC). Thus, it is possible that they have resistance genes not to individual adverse environmental factors, but to stress in general.

## Conclusions

The identification of the trace elements tolerant strains from urban sewage sludge showed that strains belong to the different species of gram-positive (*Rhodococcus qingshengii)* and gram-negative (*Serratia fonticola*, *Pseudomonas fragi*, *Stenotrophomonas maltophilia*, *Pseudomonas extremaustralis*, *Pseudomonas cedrina*, *Serratia liquefaciens* and *Citrobacter freundii*) bacteria. The metal tolerance bacterial strains correlated with the maximum content of metal(loids) in their habitat in a substantial number of cases. Our data demonstrate that the isolated trace element tolerant bacteria are, for the most part, not extreme in relation to basic environmental factors, such as temperature, pH and salinity. Thus, it can be assumed, that adaptation to chemical pollutants is a specific, rather than a stressor, mechanism. Wastewater treatment plants and sewage sludge can be hotspots for heavy metals and metalloids resistant genes and for the spread of bacteria into the environment objects. The presence of metal(loids) tolerant bacteria also increases the potential risk of gene transfer to non-resistant bacteria. The problem of disposal of sewage sludge is largely determined by the microbiological processes of decomposition of organic matter and the transformation and detoxification of pollutants that occur in them. The composition of the microbial community in sewage sludge must be adapted to extreme habitat conditions, primarily to high concentrations of trace elements and antibiotics. Thus, the microbial community present in sewage sludge especially metal(loids) tolerant can be used to create modern technologies for the effective mineralization of sewage sludge and for the decontamination of industrial and domestic wastewater.

## Materials and methods

### Sample collection and chemical analysis

In June 2022 different age’s mixed SS samples from Tula`s sewage treatment plant (capacity of 450·10^3^ m^3^/day) were taken from the silt storage sites for chemical and microbiological analysis.

The age of the selected sludge (according to the documentation of the management of the treatment facilities of the city of Tula) was 1 month, 6 months, 1 year (12 month), 5 years (about 60 month). Fresh sludge, just unloaded from the digester after dehydration, was also collected. From the sites for storing sludge of the appropriate age, using a special sampler from a layer of 20 cm using the envelope method (from five points), point samples weighing about 200 g were taken. By mixing the point samples, a combined sample was formed, which was examined. In order to prevent secondary contamination, samples were taken under aseptic conditions: with a sterile instrument, mixed on a sterile surface, and placed in a sterile plastic container. Samples sampled for chemical analysis were air dried to constant mass. The salts, acidity, content of nitrogen, carbon and hydrogen, and the concentration of total trace elements were determined in the collected SS samples.

Determination of the concentration of organic carbon (C_org_), total nitrogen (N), sulfur (S) and hydrogen (H) in SS samples was performed using an automatic HCNS analyzer Elementar Vario El III (Germany) at the Center of Common Facilities of the Institute of Physicochemical and Biological Problems in Soil Science of Russian Academy Sciences. The total concentration of metals and metalloids in the solutions obtained after microwave acid decomposition of SS in a mixture of HNO_3_ + HClO_4_ was measured by atomic absorption spectrometer with flame atomization of samples Analytik Jena contrAA® 800 (Germany) at the Laboratory of Biogeochemistry, Tula State Lev Tolstoy Pedagogical University, Tula, Russian Federation.

### Bacteria cultivation

Bacterial strains were isolated from the collected different ages SS samples, extracts were diluted 7 ten-fold dilutions by physiological solution and inoculated on the LB agar medium with following content: yeast extract—5 g/L, tryptone—10 g/L, NaCl—10 g/L, agar—15 g/L. Amount of colony-forming units (CFU) were determined as described in Standard Methods for Examination of Water and Wastewater (Standard Methods… 2022). The 100 µL of bacterial cell suspension was distributed over the surface of the agar medium in Petri dishes by a glass spatula. Dishes were placed for 24–48 h at 28℃ in a thermostat. The amount of CFU was estimated by direct counting of colonies and expressed as a quantity of bacteria in 1 g of the SS sample. Microorganisms were tested for tolerance to high concentrations of six trace elements: Co, Ni, Cu, Zn, Cd and Pb. The elements stock solutions were prepared from nitrate salts of the elements as described by Cai et al. (2014). The 100 µL of the bacterial suspension by a sterilized pipet was spread on Petri dishes with twice diluted LB medium (to prevent of trace elements complexes precipitation) with metal concentrations: 2 mmol (low tolerance), 3 mmol (middle tolerance), 5 mmol (high tolerance). Bacteria resistant to heavy metals and metalloids were isolated from SS by direct seeding on selective media. The study of bacterial resistance to six trace elements (Co, Ni, Cu, Zn, Cd, Pb) was carried out by visual assessment of their growth on a medium containing water-soluble salts of these metals at concentrations of 2 mmol (low resistance), 3 mmol (medium resistance), 5 mmol (high resistance). After incubation of dishes at 28℃ for 24 h in an incubator the bacterial colonies with different morphology, including size, edge appearance and color of colony, were visually assessed for isolation and purification.

The marked colonies were seeded by glass stick in Petri dishes with LB medium containing metal concentration 2, 3, and 5 by mmol/l. The Petri dishes were incubated in a thermostat at conditions described above, and the sowing and incubation were repeated until a single specific colony was formed. Bacterial strains were harvested from the LB medium and stored with sterilized glycerol at 5℃. From the isolated bacteria, 12 strains with different phenotype properties were selected based on their tolerance to two or three trace elements simultaneously in concentrations 3 and 5 mmol. Genomic DNA was isolated from the chosen tolerant bacterial strains using the Quick-DNA Miniprep Kit (Zymo Research, USA).

### Sequencing and identification of bacterial isolates

The isolated bacterial strains were identified using polymerase chain reaction (PCR) analysis of target genes and sequenced using Sanger sequencing method. The isolated DNA was used to amplification of the 16S rRNA gene. PCR was carried out with the two primers: forward - 27f 5'-AGAGTTTGATCCTGGCTCAG-3' and reverse - 1492r 5'-GGTTACCTTGTTACGACTT-3'. Amplification of the 16S rRNA gene was performed on GeneAmp PCR System 9700 device (Applied Biosystems, Foster City, CA, USA) at the following parameters: primary denaturation: 95℃ for 5 min; then 30 cycles: 95℃ for 30 s, 55℃ for 30 s (temperature of primer annealing), 72℃ for 40 s; final elongation stage—72℃ for 5 min.

The PCR product size was 1465 bp. The products of reaction were divided by electrophoresis in agarose gel (1.0%) at a voltage of 10 V/cm. Gel staining was performed by ethidium bromide solution (5 µg/mL) and photographed under UV light by a gel documentation system (Gel DocTMXR, Bio-Rad, Hercules, USA). PCR components, taqDNApolymerase, concentrated electrophoresis buffer, and GeneRuler 1kb DNA Ladder marker (SM0311) were produced by Fermentas (Lithuania). Products of PCR were purified by DNA Clean & Concentrator (Zymo Research, USA) according to the instructions of manufacturer.

Sequencing of the 16S rRNA gene of the bacterial strains was performed with PCR products using the described above primers. Sequencing was carried out on an automatic sequencer ABI Prism 373 3130XL (Applied Biosystems, USA). Preliminary phylogenetic screening for the similarity of nucleotide sequences of the 16S rRNA gene was received from the GenBank database of National Center for Biotechnology Information (NCBI) using the Basic Local Alignment Search Tool (BLAST) software package. To more precise study of the phylogenetic position of the investigated strain, the sub-sequent 16S rRNA gene sequence was aligned with the corresponding sequences of the closest bacterial species using the CLUSTAL W program.

### Determination of optimal environmental conditions for the growth of strains

To study the optimal growth temperatures, the isolated strains were transferred by the replica method onto LB agar medium and grown in a thermostat at different temperatures: 7, 15, 24, 30, 37, 42°C for 2–7 days, depending on temperature. The incubation time depended on the temperature, since at low temperatures strains grow longer. At temperatures of 24, 30, 37 and 42°C they were incubated for 48 hours. At a temperature of 15°C - 5 days and at 7°C - 7 days. The growth of the strains was assessed by the presence of colonies on the plate.

To determine the optimal pH for bacterial growth, individual colonies of strains from the agar medium were inoculated in test tubes with 5 ml of liquid LB medium and cultivated at 28°C for 18 h on a shaker at 160 cycles/min. Then the strains were inoculated into test tubes with LB medium with different pH values - from 4 to 9 with a step of 1 unit. The tubes were cultured at 28°C on a shaker at 160 cycles/min for 18 h. Next, the growth of strains was assessed by the optical density (OD) of the culture on a Shimadzu UV-160A spectrophotometer at λ = 260 nm.

The growth of strains at different concentrations of NaCl in the medium was assessed as follows: the isolated strains were transferred by the replica method to LB agar medium with different NaCl salt concentrations, % (1, 2, 3, 4, 5, 6, 7, 8, 9, 10). The dishes were incubated in a thermostat at 28°C for 2 days. The growth of strains was assessed by the presence of colonies on a Petri dish.

## Data Availability

The data that support the findings of this study are not openly available due to reasons of sensitivity and are available from the corresponding author upon reasonable request. Data are located in controlled access data storage at the Tula State Lev Tolstoy Pedagogical University.

## Abbreviations

SS: Sewage sludge
CFU: Colony-forming units
PCR: Polymerase chain reaction
MPC: Maximum permissible concentration
